# The Association Between Serum Copper Levels and Proteomics in Mild Cognitive Impairment

**DOI:** 10.3390/nu18081171

**Published:** 2026-04-08

**Authors:** Rachaya Rattanakarun, Prapimporn Chattranukulchai Shantavasinkul, Pirada Witoonpanich, Sittiruk Roytrakul, Jintana Sirivarasai

**Affiliations:** 1 Doctor of Philosophy Program in Nutrition, Faculty of Medicine Ramathibodi Hospital and Institute of Nutrition, Mahidol University, Bangkok 10400, Thailand; rachaya.rattanakarun@gmail.com; 2 Division of Nutrition and Biochemical Medicine, Department of Medicine, Faculty of Medicine Ramathibodi Hospital, Mahidol University, Bangkok 10400, Thailand; sprapimporn@gmail.com; 3 Division of Neurology, Department of Medicine, Faculty of Medicine Ramathibodi Hospital, Mahidol University, Bangkok 10400, Thailand; piradaw@gmail.com; 4 National Center for Genetic Engineering and Biotechnology (BIOTEC), National Science and Technology Development Agency, Pathum Thani 12120, Thailand; sittiruk@biotec.or.th; 5 Nutrition Unit, Faculty of Medicine Ramathibodi Hospital, Mahidol University, Bangkok 10400, Thailand

**Keywords:** mild cognitive impairment, serum proteomics, metal homeostasis, non-ceruloplasmin-bound copper, complement and coagulation cascades

## Abstract

Background/Objectives: Trace metal homeostasis is regulated by nutritional status and is crucial for maintaining redox balance, vascular function, and neuroinflammation. Dysregulation of systemic copper (Cu) metabolism, especially an elevated level of non-ceruloplasmin-bound copper (NCC), has been linked to oxidative stress and early cognitive decline. However, the nutritional and molecular pathways that connect Cu imbalance to mild cognitive impairment (MCI) are not well understood. Methods: We compared the serum Cu and zinc levels of individuals with normal cognition (NC; *n* = 116) and MCI (*n* = 184). An exploratory serum proteomic analysis using pooled samples was conducted to investigate patterns related to Cu dysregulation. We identified proteins using pattern correlation analysis and then performed a protein–protein interaction analysis using STRING and functional annotation and biological and Kyoto Encyclopedia of Genes and Genomes pathways. Results: The individuals with MCI had higher NCC levels than those with NC, indicating disrupted Cu metabolism influenced by nutrition and metabolism. The proteomic analysis revealed changes in proteins related to lipid transport, metal balance, and inflammation, including transthyretin, transferrin, apolipoprotein A-I, alpha-1 antitrypsin, antithrombin III, and alpha-2-macroglobulin, which respond to oxidative stress and vascular injury. Conclusions: In this cross-sectional analysis of baseline data, NCC levels were associated with cognitive status and specific circulating proteomic profiles. These findings suggest a potential relationship between copper-related biomarkers and mild cognitive impairment; however, longitudinal studies are required to clarify temporal relationships and potential mechanistic pathways.

## 1. Introduction

Trace elements, including iron (Fe), zinc (Zn), copper (Cu), selenium, and manganese, play important roles in neuronal function, as they are critical to various biological processes and molecular mechanisms [1,2]. Cu is a significant dietary cofactor of enzymes that support neuroprotection. For example, it is essential for the activity of superoxide dismutase (SOD), which effectively mitigates oxidative stress in neurons [3]. Additionally, it is crucial for cytochrome c oxidase activity, a key enzyme in mitochondrial oxidative phosphorylation, which facilitates energy production in neurons [4]. Furthermore, the Cu-binding protein ceruloplasmin (CP) functions as a serum ferroxidase, oxidizing toxic ferrous ions into ferric ions, which can then be effectively transported by transferrin [5]. Cu dyshomeostasis can lead to the development of neurodegenerative diseases by inducing oxidative stress, neuronal loss, protein misfolding, neurotransmitter synthesis, synaptic and network activity, and neuroinflammation [6].

In a healthy state, CP-bound Cu accounts for 85–95% of the Cu in the body. CP-bound Cu is nonreactive and crucial for Fe metabolism and antioxidant defense, as CP prevents cellular oxidative damage [2]. In contrast, elevated levels of non-CP-bound Cu (NCC) are linked to neurotoxicity; it can easily cross the blood–brain barrier (BBB) and consequently generate reactive hydroxyl radicals via Fenton-like reactions [6]. Furthermore, excess NCC can induce amyloid-beta (Aβ) misfolding and tau hyperphosphorylation, which are key causative factors of Alzheimer’s disease (AD) and mild cognitive impairment (MCI) [7,8,9]. Despite these important findings, few studies have specifically explored the role that Cu plays in individuals with MCI.

The current diagnostic methodologies used to confirm cognitive impairment involve obtaining cerebrospinal fluid (CSF) via an invasive procedure (to test for biomarkers) and expensive neuroimaging techniques, which present challenges for clinical and epidemiological applications [10,11]. However, technological advances have led to the identification of potential blood-based biomarkers of MCI, which could facilitate its diagnosis and enhance our understanding of its complex underlying mechanisms [12]. More specifically, the use of multiplex proteomics has uncovered molecules in CSF and plasma that are linked to early AD [13]. These significant findings suggest that monitoring the levels of several types of molecules, including immune markers, phospholipids, and angiogenic proteins, may provide complementary or alternative insights into AD to those gained from tracking conventional measures (i.e., Aβ and tau) [13]. A recent proteomic analysis identified 48 proteins linked to AD (e.g., CEND1, GFAP, NEFL, and SYT1) that can predict the risk and progression of AD, with their levels rising 15 years before diagnosis [14]. In another study, the serum proteomes of 20 Egyptians with dementia and 10 cognitively unimpaired controls were analyzed using mass spectrometry (MS) [15]. The results showed that serine protease inhibitors and immunoglobulin family proteins were downregulated, while apolipoprotein (Apo) A-II was upregulated in dementia patients. In addition, these proteins were linked to inflammation, complement activation, and lipid metabolism pathways.

Proteomic profiling is a highly promising strategy for identifying Cu-related biomarkers that will significantly advance the early detection and diagnosis of dementia, especially AD [16]. A recent study established that the dysregulation of proteins associated with Cu metabolism, including COX11, LDHA, ATOX1, SCO1, and SOD1, could be a blood-based biomarker for the early diagnosis of AD [16]. In another study, which was conducted with transgenic mice, a proteomic analysis indicated that complexin-1 and complexin-2 are the key molecules involved in chronic Cu exposure-aggravated memory impairment in AD [17].

Therefore, the aim of this study was to identify the molecular signatures linked to the high Cu levels found in individuals with MCI by conducting an in-depth serum proteomic analysis. By advancing beyond the measurement of Cu levels alone and employing a systems biology approach, we sought to fill an important gap in the literature. The findings of this study will enhance our understanding of metal–protein interactions and their implications for cognitive health.

## 2. Materials and Methods

### 2.1. Participants

This study was a cross-sectional component of the primary prospective cohort study titled “Holistic Approach to Alzheimer’s Disease in Thai People (HADThai Study),” conducted between June 2018 and March 2023 across three medical institutions in Bangkok, Thailand: Chulabhorn Hospital, Chulabhorn Royal Academy; King Chulalongkorn Memorial Hospital, Thai Red Cross Society; and Ramathibodi Hospital, Mahidol University. The participants were aged 55–80 years and comprised individuals with either normal cognition (NC) or MCI. The participants were followed up every three months for one year. At baseline, comprehensive demographic data were collected on characteristics such as age, sex, education level, self-reported history of smoking and alcohol consumption, medical comorbidities, and vitamin supplementation. Cognitive function and body composition analyses were performed at baseline, six months, and 12 months, while blood biochemistry parameters were assessed at baseline and 12 months. In this analysis, baseline clinical data were exclusively used to evaluate the associations between serum Cu levels, proteome profiles, and cognitive function.

Exclusion criteria were established to ensure participant safety and the integrity of the research findings. Individuals with a history of neurological disorders—including ischemic or hemorrhagic stroke, seizures, Parkinson’s disease, and amyotrophic lateral sclerosis—were not eligible for participation. Additionally, individuals with depression, advanced kidney disease (i.e., estimated glomerular filtration rate < 30 mL/min/1.73 m^2^), or cirrhosis classified as Child–Pugh class C were excluded due to the potential complications associated with these conditions. Individuals with poorly controlled diabetes, indicated by a hemoglobin A1c (HbA1c) level > 7%, and with a prior history of cancer, HIV infection, syphilis, or deficiencies in essential nutrients (e.g., vitamin B12 or folate) were also disqualified from the study. Before enrollment, all potential participants underwent comprehensive brain magnetic resonance imaging (MRI). Those who exhibited any signs of vascular dementia, significant white matter lesions graded III or IV, focal brain lesions, intracranial or extracranial masses, or evidence of brain infarction or hemorrhage were excluded to safeguard against any underlying neurological complications that could affect the study’s outcomes.

The parent study was approved by the Human Research Ethics Committees of Chulabhorn Hospital, King Chulalongkorn Memorial Hospital, and the Faculty of Medicine at Ramathibodi Hospital. Comprehensive explanations of the study protocol were provided to all participants and/or their caregivers, who were afforded the opportunity to ask questions. Prior to enrollment, written informed consent was obtained from all participants or their caregivers. The study was conducted in accordance with the principles established in the Declaration of Helsinki.

### 2.2. Cognitive Function Test and Diagnosis of MCI

Cognitive function was assessed using the Montreal Cognitive Assessment (MoCA) or the MoCA-B for those with less than four years of education [18,19]. The MoCA is a valuable screening tool for MCI, as it can be used to evaluate various cognitive domains, including memory, visuospatial abilities, executive functions, naming, attention, abstraction, language, and orientation. MoCA scores range from 0 to 30, with higher scores indicating better cognitive function. A cutoff score of 25 on both the MoCA and MoCA-B was employed to identify participants with MCI. The Clinical Dementia Rating (CDR) scale was also utilized [20,21]. Depression was assessed using the Thai version of the 30-item Geriatric Depression Scale (TGDS-30) [22]. The participants were classified as having either NC or MCI based on the modified Peterson criteria [23]. The classification was performed by clinical experts in dementia, including psychiatrists and neurologists. The classification was based on a comprehensive clinical assessment that included the participation of both individuals and their caregivers. The participants who met the following criteria were deemed to have NC: a satisfactory MoCA/MoCA-B score, a CDR of 0, no signs of active depression (i.e., TGDS-30 score < 10), and no other psychiatric disorders. Conversely, the participants who met the following criteria were deemed to have MCI: a MoCA/MoCA-B score < 25 and a CDR of 0.5.

### 2.3. Blood Chemistry Measurements

Following standardized fractionation protocols, blood samples were partitioned and cryopreserved at −80 °C until biochemical assessment. Fasting plasma glucose (FPG) was quantified via photometric and potentiometric assays using an Alinity C analyzer (Abbott, Chicago, IL, USA). HbA1c levels were determined via photometric transmission (Tina-quant, Roche, Mannheim, Germany), while serum insulin and *C*-peptide concentrations were measured by chemiluminescent microparticle immunoassay on an Alinity I platform (Abbott, Wiesbaden, Germany). To estimate systemic insulin resistance, the homeostasis model assessment for insulin resistance (HOMA-IR) index was derived from the FPG and insulin values according to the HOMA2 test.

### 2.4. Measurement of Serum Cu, Zn, and CP

Serum Cu concentrations were measured using a PinAAcle 900T Graphite Furnace Atomic Absorption Spectrometer (PerkinElmer, Waltham, MA, USA). Serum Zn levels were determined using an AAnalyst 400 Atomic Flame Absorption Spectrometer (PerkinElmer, Waltham, MA, USA). CP levels were determined using an automated Abbott Architect instrument (Abbott Laboratories, Abbott Park, IL, USA) and the immunoturbidimetric method. These assessments were performed in the Department of Pathology at the Faculty of Medicine, Ramathibodi Hospital, Mahidol University. This department actively participates in the periodic external quality control assessment program established by the German Society of Occupational Medicine and Environmental Medicine e.V., ensuring the accuracy and reliability of laboratory test results.

The concentration of NCC was calculated from the serum Cu (µg/dL) and serum CP (mg/dL) values using the following standard formula [24]:

NCC (µg/dL) = serum Cu − (3 × serum CP)

### 2.5. Anthropometric Measurements

Height, weight, and waist circumference were measured using standardized clinical protocols. Body mass index (BMI; kg/m^2^) was calculated by dividing the weight (kg) by the square of the height (m). Waist circumference was measured at the horizontal plane midway between the inferior margin of the last rib and the iliac crest using a calibrated, non-stretchable tape to ensure precision and reproducibility.

### 2.6. Proteomic Analysis

For proteomic analysis, a pooled-sample design with biological replication was applied. Within each study group (normal cognitive function, *n* = 116; and mild cognitive impairment [MCI], *n* = 184), participants were stratified by sex and age and randomly assigned into three independent subsets to achieve broadly comparable distributions of key demographic characteristics across pools. In the normal cognitive group, three pooled samples were generated, each consisting of serum from approximately 38–39 individuals. In the MCI group, three pooled samples were generated, each consisting of serum from approximately 61–62 individuals. Equal volumes of serum from individuals within each subset were combined to generate the pooled samples. No individual sample was included in more than one pool. Each pooled sample was analyzed separately using liquid chromatography–tandem mass spectrometry (LC–MS/MS). Protein identification and quantification were performed using standard proteomic data processing workflows. Differential protein expression between groups was evaluated using predefined statistical criteria, including fold-change thresholds and significance testing, and visualized using volcano plot analysis.

In addition, subgroup analyses were performed by categorizing participants within each group into tertiles based on serum copper levels. Within each tertile, equal volumes of serum from individuals were pooled to generate representative samples (approximately 38–39 individuals per tertile in the normal group and 61–62 individuals per tertile in the MCI group). These tertile-based pooled samples were analyzed to explore proteomic variation across copper levels within each phenotypic group. This design enabled the assessment of group-level proteomic differences while incorporating biological variability across independent pooled samples and exploring copper-related proteomic patterns.

The proteomic analysis was performed using LC-MS/MS as previously described [25]. Pooled protein samples (20 µg) obtained from the serum of participants in the NC and MCI groups were analyzed. The protein content was quantified using the Lowry method [26] and standardized in-gel digestion. MS data were analyzed using the MaxQuant platform (v1.6.1.12) with the following search parameters: trypsin specificity, a maximum of two missed cleavages, and a mass tolerance of 20 ppm. Fixed modifications included cysteine carbamidomethylation, while methionine oxidation and *N*-terminal acetylation were designated as variable modifications. To ensure high-confidence identification, proteins were filtered to include only those with a minimum peptide length of seven amino acids and a requirement of at least two peptides (one unique) per protein.

Venn diagrams were generated to comparatively analyze the distribution of the identified proteins in different study groups [27]. For quantitative analysis and biomarker discovery, we used the MetaboAnalyst platform (v6.0; https://www.metaboanalyst.ca, accessed on 20 May 2025) to conduct high-dimensional statistical analyses. A volcano plot was utilized to integrate the magnitude of change (fold change [FC]) and statistical significance (*t*-tests) data. Proteins were deemed significant if they met the criteria of a *p*-value < 0.05 and an FC > 1.5. We calculated the variable importance in projection (VIP) scores using partial least squares discriminant analysis to rank proteins by their contribution to distinguishing between study groups. A VIP score > 1.0 was used to identify key protein drivers associated with cognitive impairment. We conducted pattern correlation analysis to identify proteins that followed specific clinical trends, specifically those that decreased linearly as serum Cu levels dropped and cognitive scores declined. For this, we performed a template-based correlation analysis.

We conducted a comprehensive functional classification to enhance our understanding of the serum proteome. We used the PANTHER Classification System (www.pantherdb.org) to categorize the proteins, which allowed us to map the differentially expressed proteins (DEPs) to their associated biological processes, molecular functions, and cellular components. To visualize the interactome, we employed the STRING database (https://string-db.org/, accessed on 20 May 2025). We created a network of significant proteins using a high-confidence interaction score of 0.700. This approach not only facilitated the mapping of protein interactions but also provided valuable insights into reactome pathway enrichment. We performed separate enrichment analyses of the DEPs identified in comparisons of the NC and MCI groups, specifically within each serum Cu tertile. The threshold for statistical significance was set at a false discovery rate (FDR)-adjusted *p*-value of <0.05. Using this method, we tracked the biological mechanisms that become active or exhibit disruption as Cu levels transition from low to high. The results are presented in Lollipop charts, with redundant Gene Ontology terms grouped into overarching biological themes. This format allows a clear view of the significant patterns emerging from our analysis to be obtained.

### 2.7. Statistical Analysis

Statistical analyses were performed using SPSS (v23). Continuous variables are presented as mean ± standard deviation (SD), while categorical variables are expressed as frequencies and percentages. The distribution of continuous variables was assessed for normality using the Shapiro–Wilk test prior to statistical analysis. Variables with approximately normal distributions were compared between groups using independent-sample *t*-tests. Categorical variables were analyzed using the chi-square test. To evaluate the association between clinical and biochemical variables and the risk of mild cognitive impairment (MCI), univariate logistic regression analyses were performed, and odds ratios (ORs) with 95% confidence intervals (CIs) were calculated. Variables with statistical significance or clinical relevance were subsequently included in multivariable logistic regression models to identify independent predictors of MCI, with adjustment for potential confounders including age, sex education and BMI. A *p*-value < 0.05 was considered statistically significant.

Receiver operating characteristic (ROC) curve analysis was used to assess the diagnostic performance of potential biochemical markers and clinical covariates in identifying specific disease stages. The area under the curve (AUC) was the main metric used to evaluate accuracy. For statistically significant variables (*p* < 0.05), the optimal diagnostic cutoff was determined using the Youden Index, which maximizes the difference between true positive and false positive rates, calculated with the formula: J = (Sensitivity + Specificity) − 1. Cutoff values were not established for variables with AUC values that lacked statistical significance (*p* > 0.05), which indicated insufficient discriminatory power. Although age, education, BMI, and MoCA score were statistically significant, they were regarded as confounding covariates, and their optimal cutoff values were withheld to focus on the predictive value of novel biochemical indicators.

## 3. Results

This study included 300 participants who were divided into two groups: the NC group (*n* = 116) and the MCI group (*n* = 184) (Table 1). The MCI group had a lower cognitive test score (21.91 vs. 26.84, *p* < 0.001) and was older (67.03 vs. 64.24 years, *p* < 0.001), with 46% of its members aged 65 or older compared to 65% of the members of the NC group. The members of the MCI group had fewer years of education (14.14 vs. 15.82 years, *p* < 0.001) and a higher average BMI (24.40 vs. 23.10 kg/m^2^, *p* = 0.004). The MCI group exhibited higher levels of serum NCC (24.15 vs. 19.47 µg/dL, *p* = 0.004), Zn (91.18 vs. 87.69 µg/dL, *p* = 0.015), and *C*-peptide (2.12 vs. 1.88 ng/mL, *p* = 0.005). The NCC-to-Zn ratio was also elevated in the MCI group (0.26 vs. 0.22, *p* = 0.017). Other factors, including lipid profiles and glucose regulation, showed no significant differences between the groups. The two groups had similar total CP levels (*p* = 0.251).

The associations between clinical and biochemical variables and Mild Cognitive Impairment (MCI) are shown in Table 2. Univariate logistic regression analysis revealed significant associations with MCI for several variables: age (OR = 1.101, *p* < 0.001), years of education (OR = 0.886, *p* < 0.001), BMI (OR = 1.105, *p* = 0.003), systolic blood pressure (OR = 1.095, *p* = 0.008), serum neurotensin (NCC) (OR = 1.128, *p* < 0.001), serum zinc (OR = 1.025, *p* = 0.016), ceruloplasmin (OR = 1.393, *p* < 0.001), *C*-peptide (OR = 1.574, *p* = 0.006), and the NCC to zinc ratio (OR = 1.929, *p* < 0.001).

In multivariable logistic regression, adjusted for age and sex, NCC remained significantly associated with MCI (adjusted OR = 1.138, *p* < 0.001). Age was also independently linked to MCI (adjusted OR = 1.099, *p* = 0.001), while higher educational attainment was associated with a reduced risk (adjusted OR = 0.907, *p* = 0.024). *C*-peptide (adjusted OR = 1.628, *p* = 0.072) and ceruloplasmin (adjusted OR = 0.919, *p* = 0.074) showed borderline associations, while BMI, blood pressure, serum zinc, and HbA1C were not significantly associated after adjustment.

**Figure 1:**
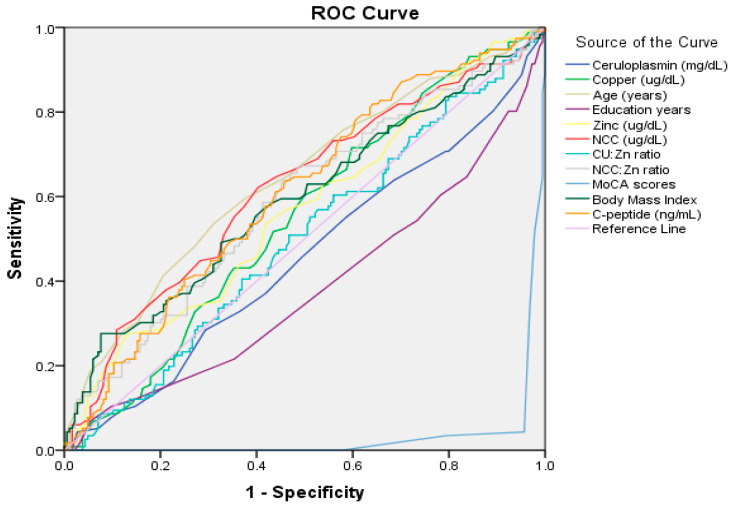
Receiver Operating Characteristic (ROC) curves evaluating the diagnostic performance of age, education, BMI, and serum biomarkers for distinguishing mild cognitive impairment from the normal cognitive group.

The diagnostic performance of various biochemical and clinical factors in identifying cognitive impairment was analyzed using ROC analysis. The results are shown in Figure 1 and Supplementary Table S1. NCC was the strongest predictor, with an AUC of 0.619 (*p* = 0.001, 95% CI; 0.553–0.685) and an optimal cutoff of ≥17.50 µg/dL, yielding 73% sensitivity and 67% specificity. *C*-peptide had an AUC of 0.608 (*p* = 0.002, 95% CI; 0.543–0.673) with a cutoff of 1.74 ng/mL, resulting in 63% sensitivity and 54% specificity. The NCC-to-Zn ratio showed an AUC of 0.590 (*p* = 0.009, 95% CI; 0.524–0.657) and a cutoff of 0.23, achieving 67% sensitivity and 51% specificity. Although Zn was significant (*p* = 0.047), it had the lowest AUC value (0.568, 95% CI; 0.501–0.635), with a cutoff of 89.50 µg/dL. Clinical factors, such as age, education, BMI, and MoCA score, were significant; however, cutoff values for these factors were not calculated since they were considered confounders in multivariate models. The low AUC values recorded for the MoCA score (0.031, *p* < 0.001, 95% CI, 0.012–0.050) and education level (0.382, *p* = 0.001, 95% CI; 0.316–0.449) suggest that individuals with lower scores for these factors have an increased likelihood of cognitive impairment.

We comprehensively analyzed the serum protein profiles of the two groups to enhance our understanding of how Cu status is related to cognitive impairment. Our results report the observed associations between serum copper levels and the identified circulating proteomic profiles in individuals with MCI and normal cognitive function. The analyses describe differential protein expression and statistical associations identified through the proteomic workflow. These results represent empirical observations derived from the dataset, whereas potential biological mechanisms underlying these associations are considered.

**Figure 2:**
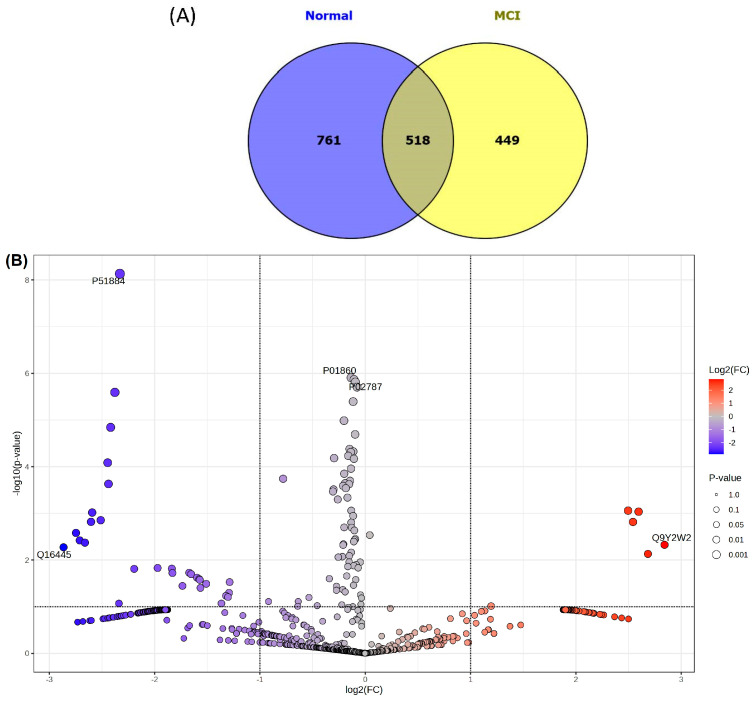
Venn diagram (**A**) and volcano plot (**B**) indicating identified proteins from normal cognitive and mild cognitive impairment groups.

As shown in Figure 2A, 1728 proteins were identified: 761 in the NC group only, 449 in the MCI group only, and 518 overlapping proteins. Functional classification of the unique proteins via PANTHER analysis revealed that the groups had distinct signaling signatures (Supplementary Table S2). While the NC group was characterized by proteins involved in cell adhesion (e.g., the Wnt pathway: CTNNB1 and CDH24) and cell cycle control (e.g., the CCKR pathway: BCL2L1 and CCND1), the MCI group demonstrated a shift toward transcriptional modulation (ELF2) and Rho GTPase-activating proteins (SRGAP2C) within the PDGF signaling framework. Notably, interleukin 6 was a consistent inflammatory marker across both cohorts.

The protein expression profiles of individuals with NC or MCI were comparatively analyzed, and the results are presented in the volcano plot shown in Figure 2B. This analysis revealed that 30 proteins were significantly downregulated and six proteins were upregulated in the MCI group. Table 3 provides a comprehensive overview of the distribution of the proteins that showed a significant trend toward downregulation in the MCI group compared to the NC group. The proteins downregulated in the MCI group exhibited log2 FC values of −1.288 to −2.865. In contrast, the proteins upregulated in the MCI group showed significantly elevated expression levels, with log2 FC values of 1.195 to 2.815. The upregulated proteins were caytazin, colipase-like protein 1, bifunctional heparan sulfate *N*-deacetylase/*N*-sulfotransferase 3 (NDST3), endothelial cell-specific molecule 1 (ESM-1), branched-chain alpha-ketoacid dehydrogenase kinase (BCKDK), and WW domain-binding protein 11. The proteins that were downregulated in the MCI group included gamma-aminobutyric acid receptor subunit alpha-6, nucleosome assembly protein 1-like 2, signal transducer and activator of transcription 3, and various complement components (e.g., C8g, C4b, and C1s).

**Figure 3:**
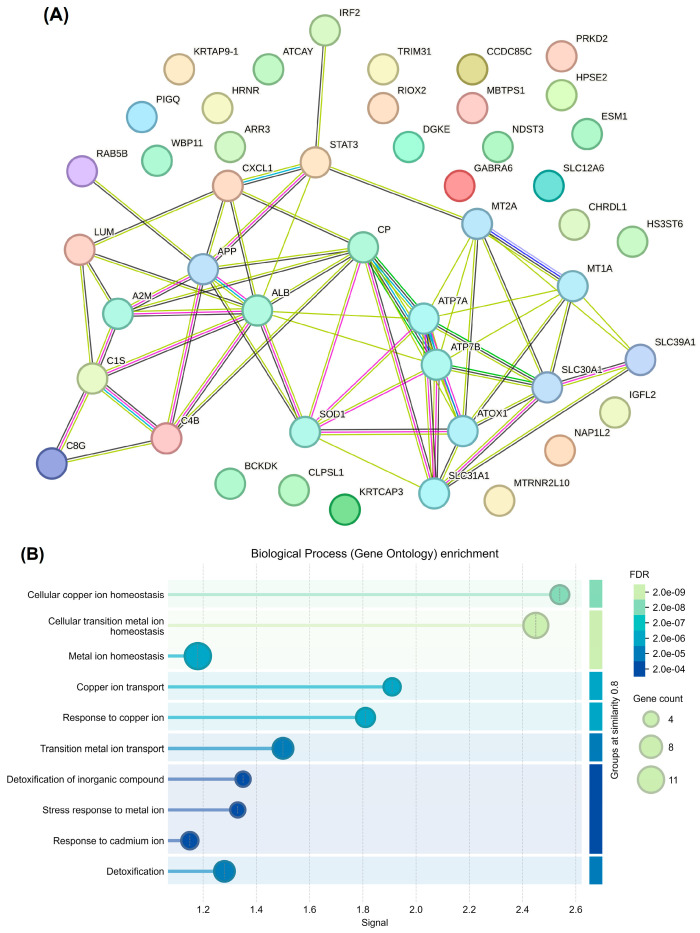
Network analysis (**A**) and enrichment pathway (**B**) of up- and down-regulated proteins compared between normal cognitive and MCI groups.

Next, we generated a STRING network to visualize the functional and physical associations among the DEPs. As shown in Figure 3A, the network featured a highly dense cluster of interactions centered around Cu homeostasis and transport proteins, including ATP7A, ATP7B, SLC31A1, SLC30A1, SLC39A1, and ATOX1. In addition, CP and albumin (ALB) were major hub nodes, bridging the Cu-transport machinery and amyloid-related signaling via amyloid precursor protein (APP). Regarding proteins involved in oxidative stress, SOD1 localized to the core Cu cluster, highlighting the link between metal ion transport and the antioxidant response. Signal transducer and activator of transcription 3 (STAT3) formed a secondary hub, with connections to immune-related proteins, such as complement C4b, C1s, and C8g, and the chemokine CXCL1.

The key biological pathways significantly affected by the proteomic differences between the study groups are illustrated in Figure 3B. The most significant pathway was cellular Cu ion homeostasis, with a remarkable FDR of 2.0 × 10^−9^. The analysis highlights the dysregulation of transition metals, particularly in relation to the cellular metal ion balance. Notably, Cu and transition metal ion transport mechanisms had high signal scores, indicating targeted disruption of metal ion movement across cellular membranes. These findings reveal important systemic responses to metal-induced toxicity. Additionally, a specific response to cadmium ions was observed that warrants further investigation into the disruption of metal ion homeostasis that affects other divalent cations.

Our functional enrichment analysis revealed that distinct biological processes were associated with different serum NCC levels. In the NC group, as the Cu level increased, there was a shift from processes related to protein stability (T1) to those involving lipids and intensive Cu detoxification (T3) (Supplementary Figure S1A–C). In contrast, the MCI group displayed a strong signature of acute-phase responses and hemostasis at lower Cu levels, and complement activation and neutral lipid metabolism processes were more prominent at the highest Cu levels (T3) (Supplementary Figure S1D–F). Importantly, Cu detoxification and humoral immune response processes were consistently detected in both groups. However, elevated Cu levels specifically intensified lipid-related and inflammatory signaling in the participants with MCI.

**Figure 4:**
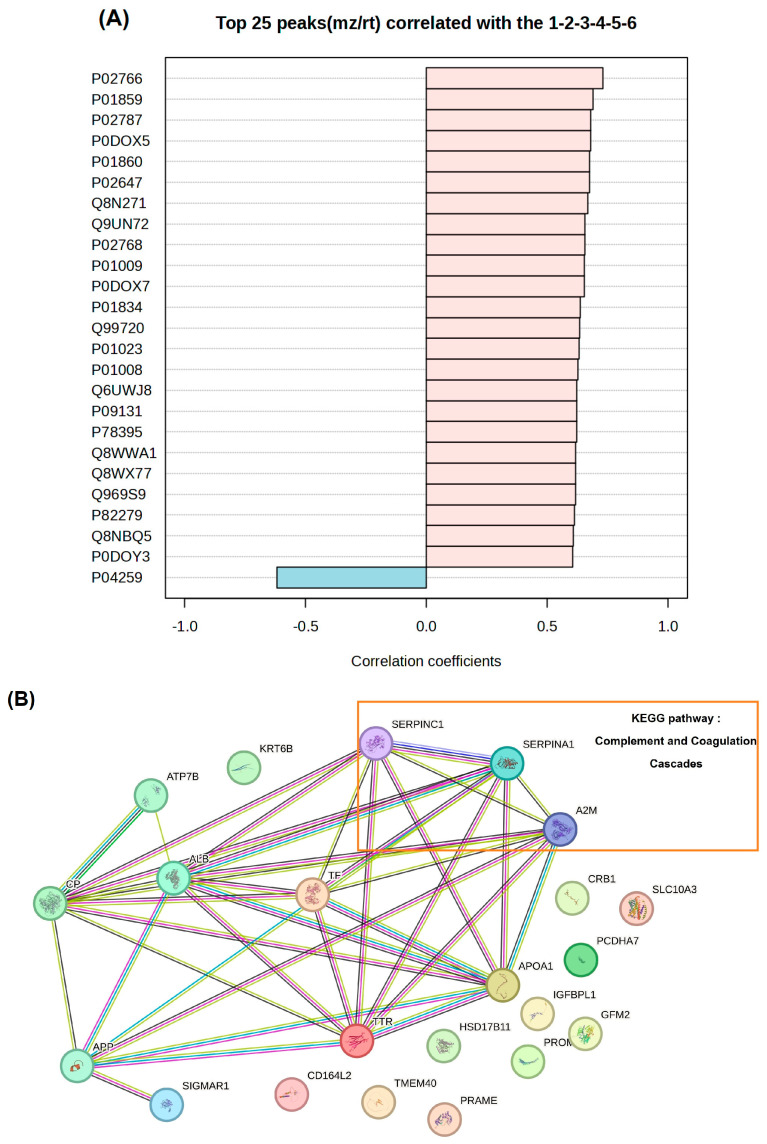
Pattern correlation analysis (**A**) and network analysis (**B**) show top 25 proteins from normal cognitive and MCI groups.

Using the MetaboAnalyst platform, we conducted a pattern correlation analysis to identify serum proteins linked to cognitive impairment associated with high Cu levels (Figure 4A). For this, we analyzed the serum Cu tertiles of the NC and MCI groups. The analysis revealed a dominant proteomic signature characterized by a positive correlation: 24 of the top 25 features that increased in abundance with cognitive impairment also increased with the Cu level. These positively correlated proteins exhibited robust correlation coefficients that ranged from approximately 0.60 to 0.75. The top-ranked proteins that drove this trend were P02766 (transthyretin), P01859 (immunoglobulin heavy constant gamma 2), and P02787 (serotransferrin/transferrin), and these are all involved in the systemic upregulation of specific transport proteins and immunoglobulins. Other notable positive correlates were lipid metabolism and acute-phase response proteins, such as P02647 (Apo A-I) and P01009 (alpha-1 antitrypsin [A1AT]). In contrast, P04259 (keratin, type II cytoskeletal 6B) was the only protein among the top 25 to exhibit a significant negative correlation (approximate coefficient: −0.60), indicating its progressive depletion as cognitive impairment rose.

We constructed a robust protein–protein interaction (PPI) network (Figure 4B) using the top 25 proteins identified in the pattern analysis. To contextualize and interpret the network in terms of Cu-related biological processes, a limited set of well-established Cu-binding and Cu-transport proteins were manually added as reference nodes (i.e., CP, ATP7B, and APP). These proteins were not identified in the proteomics dataset and were included solely to support mechanistic interpretation. Pathway mapping identified the complement and coagulation cascades as a prominent functional theme (i.e., A1AT, antithrombin III [AT III], and alpha-2-macroglobulin [A2M]). Several proteins involved in innate immune activation and hemostatic regulation were observed, suggesting coordinated changes in inflammatory and vascular-related processes.

## 4. Discussion

In this study, older age, a low education level, a high BMI, and a high *C*-peptide concentration were associated with MCI. These findings are similar to those of previous studies. A study of 482 Thai individuals with a mean age of 68.3 years found that 71.4% of older adults have MCI, with the prevalence increasing with age [28]. In that study, older adults with MCI were 1.74 times more likely to have an education level of four years or less compared to those without MCI. As individuals age, there is a notable decline in associative memory, characterized by reduced activation of the hippocampus and alterations in functional connectivity. Older adults tend to exhibit a greater reliance on cognitive control and stronger connectivity between the hippocampus and striatum. These factors may contribute to an elevated risk of MCI [29]. The Mexican Health and Aging Study identified several factors that are independently associated with MCI in older adults, including a low education level, advanced age, depression, diabetes, and residing in a rural area [30]. Another study examined the moderating effect of education (a proxy for cognitive reserve) on the relationship between the burden of white matter hyperintensities (WMH) and the transition from NC to MCI [31]. The results indicate that higher education levels correlate with a reduced WMH burden among individuals who advance to MCI. A high BMI increases the risk of developing MCI in several ways. Obesity can induce pro-inflammatory cytokines, which can weaken the BBB and cause neuronal damage, and it can also disrupt mitochondrial processes and thus impair neuronal function and contribute to cognitive decline [32]. In our study, the higher *C*-peptide levels observed in the MCI group may have been related to abnormal insulin function, which significantly impacts cerebral bioenergetics and synaptic loss [33].

In the present study, total serum copper levels were not significantly different between groups, whereas NCC was significantly associated with cognitive status. This distinction is biologically relevant, as NCC represents the loosely bound and redox-active fraction of circulating copper, which is more readily available to participate in oxidative reactions and may contribute to neuronal damage through increased oxidative stress and disruption of cellular homeostasis.

ROC curve analysis assessed the ability of NCC and related biomarkers to differentiate individuals with MCI from those with normal cognitive function. The AUC for NCC was 0.619, indicating a modest discriminative ability. Similar AUC values were found for other biomarkers, suggesting limited but noticeable differentiation. The MoCA yielded an AUC of 0.031. This apparent low value reflects the direction of classification used in the analysis, as lower MoCA scores indicate poorer cognitive performance. When interpreted accordingly, the results demonstrate that MoCA has excellent discriminative ability in distinguishing individuals with MCI from those with normal cognition. This finding underscores the practical utility of MoCA as a sensitive cognitive screening tool for the early detection of mild cognitive impairment in clinical and research settings [19]. While the NCC:Zn ratio and *C*-peptide showed significant discriminative performance, they did not demonstrate an independent association with MCI in multivariable logistic regression, implying potential confounding factors. In contrast, NCC is a more reliable independent biomarker for MCI, suggesting its value for further research and clinical use.

In alignment with previous studies, elevated serum NCC levels and cognitive impairment were found to be connected in this study. A longitudinal study of individuals with MCI found that serum NCC is a significant predictor of the progression of MCI to AD [34]. This association underscores the critical role that Cu dyshomeostasis, particularly an increase in the level of NCC, plays in cognitive decline. It is thought that higher levels of NCC influence cognitive decline due to Cu’s redox-active properties and potential toxicity, which likely arise from its capacity to induce oxidative stress and the formation of advanced glycation end-products [35]. Such processes can damage the extracellular matrix in various tissues, including brain tissue [35]. However, it is important to note that not all individuals with high Cu levels will experience cognitive decline. This is because etiological and pathophysiological factors, including genetic, environmental, and lifestyle factors, interact in complex relationships to impact the development and progression of neurodegenerative diseases [6]. Hence, there is a need for more in-depth studies on the role that NCC plays in the etiology of specific neurodegenerative diseases.

It should be noted that the proteomic analysis in this study was conducted using pooled serum samples. While this approach can facilitate the identification of group-level proteomic patterns, it limits the ability to assess inter-individual variability in protein expression. Therefore, the identified proteins should be interpreted as exploratory findings and potential candidates for further investigation rather than definitive biomarkers.

Proteins unique to the MCI group are involved in PDGF and inflammation signaling pathways (e.g., Rho GTPase-activating proteins). In the condition termed “leaky brain,” strong Rho-associated protein kinase activity has been shown to destabilize tight junction proteins in endothelial cells, thereby increasing BBB permeability [36]. Consequently, peripheral toxins and inflammatory cells can infiltrate the brain, which is a hallmark of both AD and vascular dementia. Recent studies have shown that Rho proteins play critical roles in the regulation of hippocampal long-term potentiation, which is closely related to synaptic plasticity and is considered the cellular mechanism underlying learning and memory [37]. Additionally, proto-oncogene Wnt-3, which is involved in the Wnt signaling pathway, was found to be unique to the MCI group. Dysregulation of this protein is linked to abnormalities in synaptic plasticity and neuronal function [38].

Regarding the proteins downregulated in the MCI group, Ras-related protein Rab-5B is involved in neuronal endocytosis and synaptic processes. Perturbation of Rab5 signaling has been shown to impair these processes and affect neurite morphology and axonal traffic [39]. Low levels of STAT3, another downregulated protein, can lead to increased microglial M1 polarization, which is associated with neuroinflammation and cognitive impairment [40]. Diacylglycerol kinase ε (DGKE) was also downregulated in the MCI group. This protein catalyzes the phosphorylation of diacylglycerol (DAG) to produce phosphatidic acid (PA), and both DAG and PA are key second messengers in neurons. Low DGKE levels could plausibly cause neuronal dysfunction by disrupting DAG–PKC signaling, reducing PA production, and increasing neuronal vulnerability to lipid imbalances [41,42].

Several proteins were upregulated in the MCI group, including BCKDK. This protein inhibits the breakdown of branched-chain amino acids (BCAAs), such as leucine, isoleucine, and valine. Hence, high levels of BCKDK can result in high levels of BCAAs, which compete with tryptophan for transport across the BBB. A lack of tryptophan can result in reduced serotonin synthesis and increased inflammation, which enhances the risk of cognitive decline and dementia [43,44]. ESM-1, or endocan, is secreted by endothelial cells and contributes to endothelial dysfunction and inflammation. It is involved in vascular permeability and leukocyte infiltration, which potentially lead to neurological disorders [45]. NDST3 modifies heparan sulfate on proteoglycans, impacting synaptic function and protein aggregation. Abnormal NDST3 levels may increase the risk of dementia by compromising the integrity of the BB and by promoting Aβ aggregation, tau pathology, synaptic dysfunction, and inflammation [46].

Our PPI network analysis showed that DEPs associated with inflammation (e.g., complement components, interferons, and chemokines) are interconnected to proteins involved in Cu homeostasis (e.g., ALB, CP, and metallothionein) and APP. Our findings reveal an integrated network of several critical pathophysiological factors, including aging, metabolic disorders, vascular damage, oxidative stress, and metal dyshomeostasis. These factors are associated with inflammatory mediators that initiate APP processing, which may lead to chronic neuroinflammation and synaptic degeneration [47,48]. Additionally, our tertile-based analysis of serum Cu levels showed enriched pathways that align with this integrated network, suggesting avenues for further research and potential therapeutic interventions.

A coherent proteomic response was observed in the pattern correlation analysis. Serum proteins that increased in abundance with cognitive impairment also increased with the Cu level. Notably, elevated serum transthyretin levels were recorded in the MCI group, suggesting a compensatory response to early amyloid pathology, which may decline as AD progresses [49]. Increased levels of transferrin reflect Fe dyshomeostasis and oxidative stress, both of which are strongly implicated in cognitive decline [50]. The increased levels of Apo A-I observed in the presence of elevated Cu in our study align with previous research showing that high Cu levels can lead to oxidative modification of Apo A-I [51]. This modification results in dysfunctional HDL and raises the risk of dementia, even though the level of circulating Apo A-I is elevated. The structural protein *KRT6B* exhibited a negative correlation with the Cu level in our study. In another study, transcriptomic analysis revealed that the *KRT6B* gene is the most highly connected gene within the AD network and that the dysregulated expression of keratin genes may play a role in the initiation and progression of dementia [52].

Furthermore, three proteins involved in complement and coagulation pathways (A1AT, AT III, and A2M) were upregulated in the MCI group. Dysregulation of these pathways has been implicated in neuroinflammation, cerebrovascular dysfunction, and cognitive impairment. In diseases of the central nervous system, including dementia and motor neuron disease, chronic complement activation is associated with glial activation and synapse and neuron loss [53,54]. Our findings indicate that an elevated serum Cu level may contribute to oxidative stress and endothelial dysfunction, which may, in turn, activate the complement and coagulation cascades. The observed increases in A1AT, AT III, and A2M likely represent a compensatory protease-inhibitory and anti-inflammatory response [55,56,57]. Such changes are consistent with early vascular–inflammatory mechanisms implicated in MCI.

Beyond the individual protein findings, the observed alterations can be integrated into broader biological pathways linking copper dysregulation to cognitive decline. Elevated levels of NCC, a redox-active fraction of circulating copper, may promote oxidative stress and disrupt multiple metabolic and cellular processes. In the context of lipid metabolism, copper-induced oxidative stress has been associated with the accumulation of diacylglycerol (DAG), potentially through dysfunction of diacylglycerol kinase epsilon (DGKE) [41,42]. In addition, oxidative stress may activate nuclear factor erythroid 2–related factor 2 (Nrf2), a key transcription factor involved in lipid homeostasis, which can upregulate lipogenic genes and contribute to altered lipid metabolism [58]. Oxidative modification of apolipoprotein A-I may further impair its interaction with high-density lipoprotein (HDL), thereby disrupting reverse cholesterol transport and exacerbating lipid dysregulation [51].

In terms of inflammatory responses, elevated activity of branched-chain α-keto acid dehydrogenase kinase (BCKDK) may reduce the catabolism of branched-chain amino acids (BCAAs), leading to their accumulation and promoting macrophage activation, oxidative stress, and chronic inflammation [43]. Concurrently, copper overload may induce mitochondrial dysfunction, triggering activation of the NLRP3 inflammasome and amplifying inflammatory signaling [59]. These processes may act synergistically, linking metabolic dysregulation with neuroinflammatory responses relevant to cognitive decline.

Furthermore, alterations in vascular-related proteins suggest a potential role of copper dysregulation in endothelial dysfunction. Oxidative stress induced by elevated copper levels may affect endothelial proteoglycans, including ESM1 and NDST3, which have been implicated in vascular pathology and atherosclerosis [45,46]. In addition, changes in complement-related proteins may reflect vascular inflammation and impaired cerebral perfusion [53,54]. Together, these interconnected pathways involving lipid metabolism, immune activation, and vascular dysfunction provide a plausible mechanistic framework linking NCC to the pathophysiology of mild cognitive impairment.

The present findings demonstrate significant associations between serum non-ceruloplasmin-bound copper (NCC) and alterations in circulating proteomic profiles in individuals with mild cognitive impairment. These results are based on observed differences in protein expression and statistical associations identified in the current dataset. However, the underlying biological mechanisms linking copper dysregulation to these proteomic changes cannot be directly established from this analysis. The potential mechanistic pathways discussed herein should therefore be interpreted as plausible hypotheses rather than definitive explanations. In addition, the findings of this study should also be interpreted in the context of the study population. Participants were recruited from hospital-based settings in Thailand, and differences in dietary patterns, environmental exposures, and genetic backgrounds across populations may influence circulating copper levels, proteomic profiles, and their associations with cognitive status. Therefore, the generalizability of the present findings to other populations may be limited. Future studies conducted in more diverse populations and settings are warranted to validate and extend these observations.

## 5. Conclusions

Our findings highlight several important aspects. We integrate clinical, biochemical, and proteomic data to explore the relationship between non-ceruloplasmin-bound copper (NCC) and cognitive status, providing a multidimensional perspective within the field of clinical nutrition. The relatively large sample size used in the primary analysis enhances the reliability of the observed associations. The application of both multivariable logistic regression and ROC analysis allows for an evaluation of NCC as a potential biomarker. Additionally, the use of proteomic profiling offers novel insights that link trace element metabolism to cognitive impairment. Together, these strengths contribute to the growing evidence regarding the role of micronutrient-related mechanisms in the early stages of cognitive decline. Several limitations of the present study should be considered when interpreting the findings. In this cross-sectional analysis of baseline data, NCC levels were associated with cognitive status and specific circulating proteomic profiles. These findings suggest a potential relationship between copper-related biomarkers and mild cognitive impairment; however, longitudinal studies are required to clarify temporal relationships and potential mechanistic pathways. This study was conducted using pooled serum samples; therefore, the identified proteins represent potential biomarker candidates and that they have not been validated at the individual level. In addition, several factors that may influence circulating copper levels and the serum proteome, including recent dietary intake, micronutrient supplementation, and systemic inflammatory status, were not fully controlled in the present study. These factors may contribute to variability in the observed biomarker profiles and should be considered in future investigations.

## Figures and Tables

**Table 1:** Characteristics of the study population.

Variables	Total	Normal	MCI	*p*-Value
	*n* = 300	*n* = 116	*n* = 184	
Age, years	65.32 ± 5.50	64.24 ± 4.96	67.03 ± 5.89	<0.001
Age groups, *n* (%)				
<65 years	140 (46.7)	41 (35.3)	99 (53.8)	0.002
≥65 years	160 (53.3)	75 (64.7)	85 (46.2)	
Gender, %				
Female	216 (72.0)	78 (67.2)	138 (75.0)	0.145
Male	84 (28.0)	38 (32.8)	46 (25.0)	
Education, years	15.17 ± 3.18	15.82 (3.35)	14.14 (4.26)	<0.001
Education, *n* (%)				
≤12 years	71 (23.7)	30 (16.3)	41 (35.3)	<0.001
>12 years	229 (76.3)	154 (83.7)	75 (64.7)	
Smoking status, %				
Non-smoker	287 (95.7)	176 (95.7)	111 (95.7)	0.988
Smoker	13 (4.3)	8 (4.3)	5 (4.3)	
Alcohol drinking, %				
Non-drinker	191 (63.7)	113 (61.4)	78 (67.2)	0.307
Drinker	109 (36.3)	71 (38.6)	38 (32.8)	
Hypertension, %	77 (25.6)	47 (25.5)	30 (25.9)	0.951
Dyslipidemia, %	103 (34.3)	58 (31.5)	45 (38.8)	0.196
Type 2 diabetes, %	15 (5)	8 (4.3)	7 (6.0)	0.514
Cardiovascular disease, %	11 (3.7)	4 (2.2)	7 (6.0)	0.083
BMI, kg/m^2^	23.60 ± 3.64	23.10 ± 3.23	24.40 ± 4.10	0.004
Waist circumference, cm	81.63 ± 9.61	80.82 ± 8.64	82.92 ± 10.90	0.081
SBP, mm Hg	129.88 ± 16.22	128.48 ± 16.21	132.09 ± 16.06	0.061
DBP, mm Hg	75.39 ± 10.17	74.57 ± 10.67	76.69 ± 9.23	0.079
MoCA scores	24.93 ± 3.19	26.84 ± 1.74	21.91 ± 2.55	<0.001
Serum copper, µg/dL	111.08 ± 18.13	110.76 ± 19.33	111.59 ± 16.12	0.702
Serum non-ceruloplasmin-bound copper (NCC), µg/dL	21.28 ± 13.78	19.47 ± 13.76	24.15 ± 13.38	0.004
Ceruloplasmin, mg/dL	29.77 ± 4.63	30.01 ± 4.54	29.38 ± 4.78	0.251
Serum zinc, µg/dL	89.04 ± 12.07	87.69 ± 11.95	91.18 ± 13.12	0.015
Serum Cu:Zn ratio	1.26 ± 0.26	1.27 ± 0.28	1.25 ± 0.24	0.571
Serum NCC:Zn ratio	0.24 ± 0.15	0.22 ± 0.15	0.26 ± 0.14	0.017
Cholesterol, mg/dL	210.96 ± 40.24	213.90 ± 39.54	206.30 ± 41.06	0.111
Triglyceride, mg/dL	104.06 ± 55.85	103.95 ± 59.86	104.25 ± 49.09	0.963
HDL-C, mg/dL	62.60 ± 15.56	62.72 ± 14.71	62.42 ± 16.88	0.873
LDL-C, mg/dL	131.26 ± 38.40	134.48 ± 38.71	126.15 ± 37.51	0.067
Insulin, µIU/mL	6.13 ± 3.44	5.87 ± 3.26	6.52 ± 3.69	0.112
*C*-peptide, ng/mL	1.98 ± 0.73	1.88 ± 0.71	2.12 ± 0.74	0.005
HOMA-IR	1.45 ± 0.56	1.40 ± 0.54	1.52 ± 0.59	0.090
Fasting plasma glucose, mg/dL	90.36 ± 10.58	89.87 ± 10.80	91.14 ± 10.22	0.313
HbA1C, %	5.68 ± 0.42	5.64 ± 0.40	5.73 ± 0.43	0.057

Data are presented as mean ± SD, or number (percent) as indicated. BMI: body mass index; Cu: copper; Cu:Zn ratio: copper/zinc ratio; DBP: diastolic blood pressure; NCC:Zn ratio: non-ceruloplasmin-bound copper/zinc ratio; HbA1C: glycated hemoglobin; HDL-C: high-density lipoprotein cholesterol; HOMA-IR: homeostatic model assessment of insulin resistance. LDL-C: low-density lipoprotein cholesterol; SBP: systolic blood pressure.

**Table 2:** Univariate and multivariate logistic regression analysis of factors associated with MCI.

Univariate Logistic Regression				Multivariate Logistic Regression		
Variables	OR	95% CI	*p*-Value	Adjusted OR *	95% CI	*p*-Value
Age (years)	1.101	1.052–1.152	<0.001	1.099	1.037–1.164	0.001
Sex (male vs. female)	1.462	0.876–2.438	0.146	1.882	0.918–3.859	0.084
Education (years)	0.886	0.829–0.946	<0.001	0.907	0.833–0.987	0.024
BMI (kg/m^2^)	1.105	1.034–1.180	0.003	1.002	0.899–1.117	0.968
SBP (mmHg)	1.095	1.024–1.171	0.008	0.977	0.974–1.020	0.786
DBP (mmHg)	1.021	0.998–1.045	0.80	1.014	0.976–1.054	0.475
Serum copper (µg/dL)	1.003	0.990–1.015	0.701	0.991	0.904–1.084	0.825
Serum NCC (µg/dL)	1.128	1.092–1.164	<0.001	1.138	1.071–1.181	<0.001
Serum zinc (µg/dL)	1.025	1.004–1.045	0.016	1.008	0.982–1.034	0.562
Ceruloplasmin (mg/dL)	1.393	1.167–1.662	<0.001	0.919	0.839–1.008	0.074
*C*-peptide (ng/mL)	1.574	1.139–2.175	0.006	1.628	0.956–2.772	0.072
HbA1C (%)	1.733	0.981–3.060	0.058	1.310	0.731–2.349	0.369

BMI, body mass index; DBP, diastolic blood pressure, NCC, non-ceruloplasmin-bound copper; OR, odds ratio; CI, confidence interval; Zn, zinc. * Adjusted OR for MCI was obtained using logistic regression analyses after controlling for age, sex education and BMI.

**Table 3:** Upregulated and downregulated proteins compared between normal and MCI groups (Data from the volcano plot with *p* < 0.001).

Protein ID	Protein Name	log2 (FC)	Protein ID	Protein Name	log2 (FC)
Downregulated Proteins (*n* = 30): Higher expression in normal cognitive group than MCI group					
Q16445	Gamma-aminobutyric acid receptor subunit alpha-6	−2.865	Q8IUF8	Ribosomal oxygenase 2	−1.837
Q9ULW6	Nucleosome assembly protein 1-like 2	−2.747	P40763	Signal transducer and activator of transcription 3	−1831
A6NKD9	Coiled-coil domain-containing protein 85C	−2.711	A8MXZ3	Keratin-associated protein 9-1	−1.736
Q8WWQ2	Inactive heparinase-2	−2.662	P0CJ77	Humanin like-10	−1.672
Q53RY4	Keratinocyte-associated protein 3	−2.604	Q9BZY9	E3 ubiquitin–protein ligase TRIM31	−1.653
P52429	Diacylglycerol kinase epsilon	−2.593	Q86YZ3	Hornerin	−1.599
Q9UHW9	Solute carrier family 12 member 6	−2.512	A0A0B4J1X5	Immunoglobulin heavy variable 3-74	−1.576
Q9BRB3	Phosphatidylinositol *N*-acetylglucosaminyltransferase subunit Q	−2.446	Q6UWQ7	Insulin growth factor-like family member 2	−1.572
P07360	Complement component C8 gamma chain	−2.438	P09871	Complement C1s subcomponent	−1.565
P61020	Ras-related protein Rab-5B	−2.418	Q9BU40	Chordin-like protein 1	−1.510
P0C0L5	Complement C4-B	−2.378	Q6DRA6	Putative histone H2B type 2-D	−1.336
Q14703	Membrane-bound transcription factor site-1 protease	−2.399	A0A0C4DH25	Immunoglobulin kappa variable 3D-20	−1.323
P51884	Lumican	−2.330	P14316	Interferon regulatory factor 2	−1.303
Q9BZL6	Serine/threonine-protein kinase D2	−2.194	P36575	Arrestin-C	−1.294
P09341	Growth-regulated alpha protein	−1.972	Q96QI5	Heparan sulfate glucosamine 3-O-sulfotransferase 6	−1.288
Upregulated Proteins (*n* = 6): Higher expression in MCI group than normal cognitive group					
Q86WG3	Caytazin	1.195			
A2RUU4	Colipase-like protein 1	2.496			
O95803	Bifunctional heparan sulfate *N*-deacetylase/*N*-sulfotranferase 3	2.542			
Q9NQ30	Endothelial cell-specific molecule 1	2.596			
O14874	Branched-chain alpha-ketoacid dehydrogenase kinase	2.684			
Q9Y2W2	WW domain-binding protein 11	2.815			

## Data Availability

The data presented in the current study are not publicly available owing to privacy and ethical restrictions. However, data are available from the corresponding authors upon reasonable request.

## Supplementary Materials

The following supporting information can be downloaded at: https://www.mdpi.com/article/10.3390/nu18081171/s1, Figure S1: Enrichment pathway of total proteins related to biological process among normal cognitive (A–C) group and MCI group (D–F) classified by serum Cu tertiles; Table S1: The optimal cut-off values and diagnostic performance metrics of various factors associated with cognitive impairment; Table S2: PANTHER Pathway Analysis for unique proteins identified in normal cognitive and mild cognitive impairment groups.
