# Supplementary material for: The Association Between Serum Copper Levels and Proteomics in Mild Cognitive Impairment
Source: Nutrients. 2026 Apr 8;18(8):1171. doi: 10.3390/nu18081171 (PMC13118556; doi:10.3390/nu18081171)
Supplement: Supplementary file 1 [file nutrients-18-01171-s001.zip › Supplementary Tables S1-S2.pdf]

Supplementary Table S1. The optimal cut-off values and diagnostic performance metrics of various factors associated with cognitive impairment.

Factors	AUC	P-value	95% Confidence Interval	Optimal Cut-off Value (Units)	Sensitivity (%)	Specificity (%)
Age (years)	0.641	<0.001	0.576-0.705	-	-	-
Education years	0.382	0.001	0.316-0.449	-	-	-
MoCA scores	0.031	<0.001	0.012-0.050	-	-	-
Body Mass Index	0.592	0.007	0.525-0.660	-	-	-
Copper (ug/dL)	0.549	0.153	0.483-0.615	-	-	-
Ceruloplasmin (mg/dL)	0.455	0.187	0.387-0.522	-	-	-
CU:Zn ratio	0.499	0.983	0.432-0.566	-	-	-
Zinc (ug/dL)	0.568	0.047	0.501-0.635	≥89.50	53	58
NCC (ug/dL)	0.619	0.001	0.553-0.685	≥17.50	73	67
NCC:Zn ratio	0.590	0.009	0.524-0.657	≥0.23	67	51
C-peptide (ng/mL)	0.608	0.002	0.543-0.673	≥1.74	63	54

Supplementary Table S2. PANTHER Pathway Analysis for unique proteins identified in normal cognitive and mild cognitive impairment groups.

PANTHER Pathway	ID proteins	Protein name
**Normal cognitive group**		
Wnt signaling pathway	P35222	Catenin beta-1
	Q86UP0	Cadherin-24
	Q9Y5E8	Protocadherin beta-15
	Q9Y5E4	Protocadherin beta-5
	Q9Y5H2	Protocadherin gamma-A11
	Q08209	Protein phosphatase 3 catalytic subunit alpha
	P49674	Casein kinase I isoform epsilon
	P54792	Putative segment polarity protein disheveled homolog DVL1P1
	Q14738	Serine_threonine-protein phosphatase 2A 56 kDa regulatory subunit delta isoform
	Q9HCS4	Transcription factor 7-like 1
	Q04725	Transducin-like enhancer protein 2
	Q9Y6F9	Protein Wnt-6
	Q12841	Follistatin-related protein 1
	Q9HCS4	Transcription factor 7-like 1
	P24385	G1_S-specific cyclin-D1
Inflammation mediated by chemokine and cytokine signaling pathway	O15530	3-phosphoinositide-dependent protein kinase 1
	O14936	Peripheral plasma membrane protein CASK
	Q13557	Calcium_calmodulin-dependent protein kinase type II subunit delta
	P41597	C-C chemokine receptor type 2
	P32302	C-X-C chemokine receptor type 5
	P21462	fMet-Leu-Phe receptor
	P25090	N-formyl peptide receptor 2
	Q6S8J3	POTE ankyrin domain family member E
	P10301	Ras-related protein R-Ras
	P42224	Signal transducer and activator of transcription 1-alpha_beta
CCKR signaling map	Q07817	Bcl-2-like protein 1
	P24385	G1_S-specific cyclin-D1
	P49674	Casein kinase I isoform epsilon
	P19875	C-X-C motif chemokine 2
	Q08209	Protein phosphatase 3 catalytic subunit alpha
	P18146	Early growth response protein 1
	P01275	Pro-glucagon
	O00255	Menin
	O15530	3-phosphoinositide-dependent protein kinase 1
	P15884	Transcription factor 4
	P35222	Catenin beta-1
**MCI group**		
Wnt signaling pathway	Q9Y2G4	Ankyrin repeat domain-containing protein 6
	P50148	Guanine nucleotide-binding protein G(q) subunit alpha
	O15379	Histone deacetylase 3
	Q99717	Mothers against decapentaplegic homolog 5
	P36896	Activin receptor type-1B

	P56703	Proto-oncogene Wnt-3
Inflammation mediated by chemokine and cytokine signaling pathway	P50148	Guanine nucleotide-binding protein G(q) subunit alpha
	P05231	Interleukin-6
	Q13153	Serine_threonine-protein kinase PAK 1
	P15056	Serine_threonine-protein kinase B-raf
	O14921	Regulator of G-protein signaling 13
	P08134	Rho-related GTP-binding protein RhoC
PDGF signaling pathway	Q15723	ETS-related transcription factor Elf-2
	P0DJJ0	SLIT-ROBO Rho GTPase-activating protein 2C
	Q53QZ3	Rho GTPase-activating protein 15
	P15056	Serine_threonine-protein kinase B-raf
	Q96S38	Ribosomal protein S6 kinase delta-1

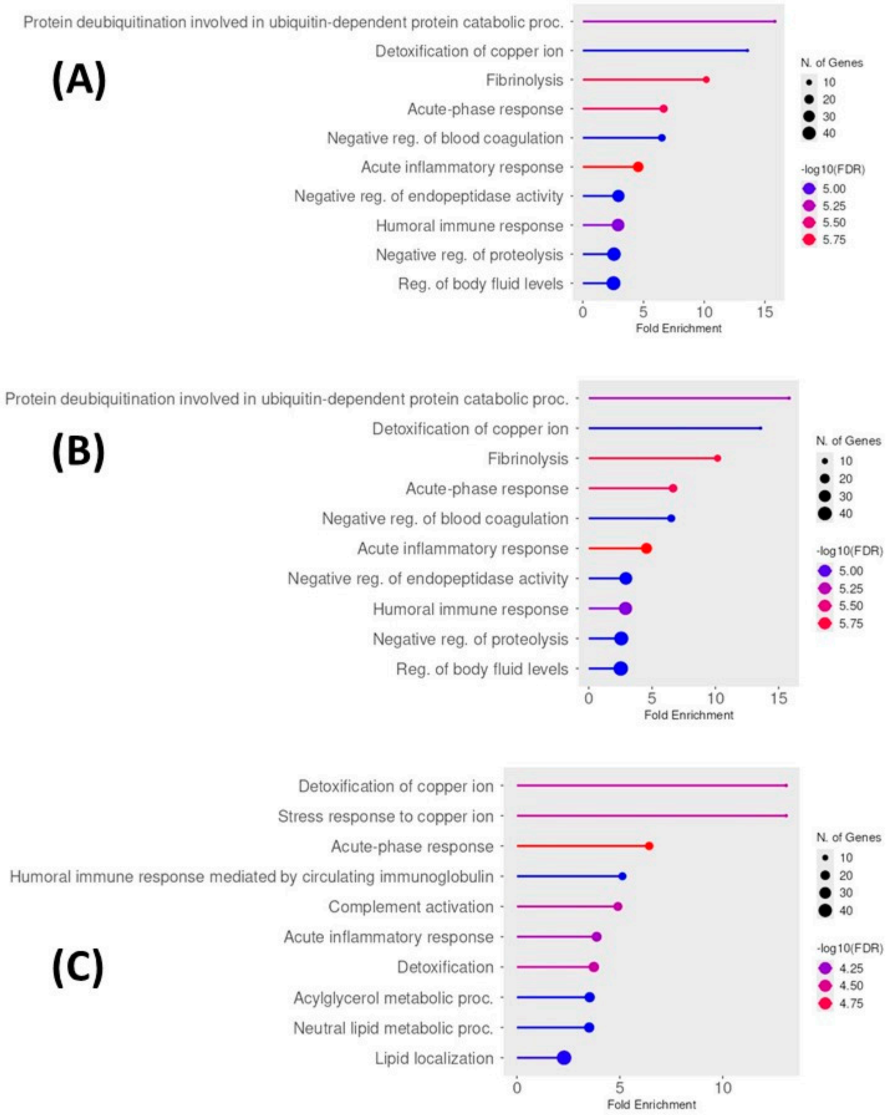
(A)
Protein deubiquitination involved in ubiquitin-dependent protein catabolic proc.
Detoxification of copper ion
Fibrinolysis
Acute-phase response
Negative reg. of blood coagulation
Acute inflammatory response
Negative reg. of endopeptidase activity
Humoral immune response
Negative reg. of proteolysis
Reg. of body fluid levels
0
5
10
15
Fold Enrichment
N. of Genes
10
20
30
40
-log10(FDR)
5.00
5.25
5.50
5.75
(B)
Protein deubiquitination involved in ubiquitin-dependent protein catabolic proc.
Detoxification of copper ion
Fibrinolysis
Acute-phase response
Negative reg. of blood coagulation
Acute inflammatory response
Negative reg. of endopeptidase activity
Humoral immune response
Negative reg. of proteolysis
Reg. of body fluid levels
0
5
10
15
Fold Enrichment
N. of Genes
10
20
30
40
-log10(FDR)
5.00
5.25
5.50
5.75
(C)
Detoxification of copper ion
Stress response to copper ion
Acute-phase response
Humoral immune response mediated by circulating immunoglobulin
Complement activation
Acute inflammatory response
Detoxification
Acylglycerol metabolic proc.
Neutral lipid metabolic proc.
Lipid localization
0
5
10
Fold Enrichment
N. of Genes
10
20
30
40
-log10(FDR)
4.25
4.50
4.75

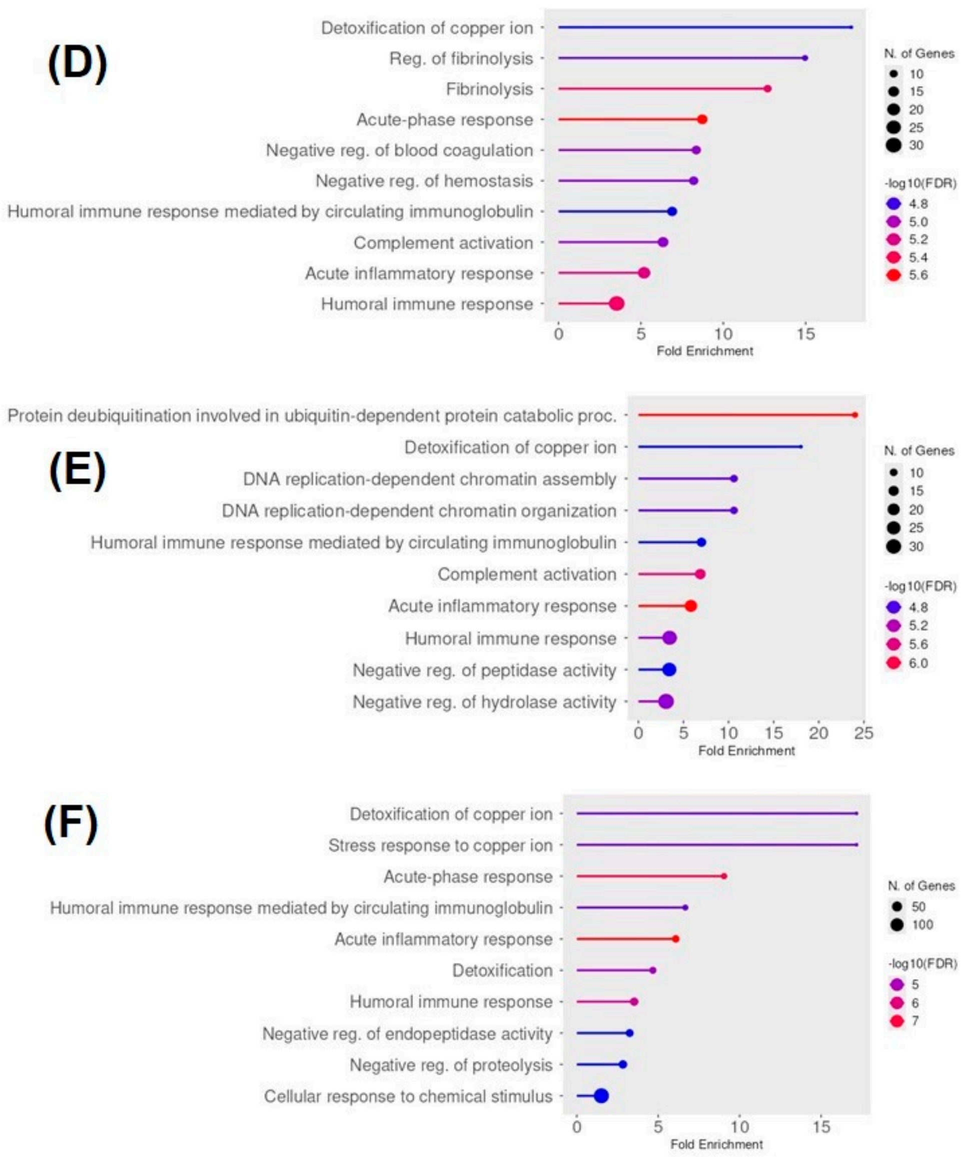

Supplementary Figure S1. Enrichment pathway of total proteins related to biological process among normal cognitive (A-C) group and MCI group (D-E) classified by serum Cu tertiles.
